# Identification of a Macrophage marker gene signature to evaluate immune infiltration and therapeutic response in hepatocellular carcinoma

**DOI:** 10.1016/j.heliyon.2024.e31881

**Published:** 2024-05-27

**Authors:** Tong Li, Xin Xu, Mengzhou Guo, Jing Guo, Kiyoko Nakayama, Zhenggang Ren, Lan Zhang

**Affiliations:** a Liver Cancer Institute & Key Laboratory of Carcinogenesis and Cancer Invasion, Zhongshan Hospital, Fudan University, Shanghai, China; b Department of Medical Oncology, Zhongshan Hospital, Fudan University, Shanghai, China; c Department of Gastroenterology, Zhongshan Hospital Xuhui Branch, Fudan University, Shanghai, China; d Department of Gastroenterology and Hepatology, Zhongshan Hospital, Fudan University, Shanghai, China

**Keywords:** Hepatocellular carcinoma (HCC), Single-cell RNA sequencing, Macrophages, Drug therapy

## Abstract

**Background:**

Only a minority of hepatocellular carcinoma (HCC) patients can benefit from systemic regimens. Macrophages, which abundantly infiltrate in HCC, could mediate tumour microenvironment remodelling and immune escape, proving to be powerful weapons in combating HCC. Thus, a deeper understanding of macrophages is necessary for improving existing antitumour treatments.

**Methods:**

With a series of bioinformatic approaches, we comprehensively explored the role of macrophage-related genes in human HCCs from multiple single-cell and bulk RNA sequencing datasets. Unsupervised clustering was performed to cluster the macrophage marker genes (MMGs). GSVA and functional enrichment analysis were used to elucidate the functional differences among the MMG-associated clusters. Subsequently, a component analysis algorithm was used to construct a Macrosig score, and the prognosis, biological characteristics, mutation profile, TME cell infiltration status and drug response of patients with different Macrosig scores were further analysed.

**Results:**

We identified 13 MMGs in 574 HCC samples, based on which three MMG-associated clusters were defined. Overall survival time, clinicopathological features and immune infiltration scores differed among the different clusters. On this basis, 12 hub genes were identified among these clusters; subsequently, a scoring system was constructed to determine the Macrosig score. Importantly, patients with low-Macrosig scores, characterized by increased immune infiltration, increased mutation frequency and increased immune checkpoint expression, including CTLA-4, LAG3, PDCD1 and TIGIT, exhibited enhanced efficacy of immunotherapy when validated in an external database. Moreover, a low-Macrosig score indicates increased sensitivity to AZD.2281, A.443654, ABT.263, ABT.888, AG.014699 and ATRA, while a high Macrosig score indicates increased sensitivity to AZD6482, AKT inhibitor VIII, AS601245, AZ628, AZD.0530 and AZD6244.

**Conclusions:**

A novel scoring system was constructed to guide more effective prognostic evaluation and tailoring therapeutic regimens for HCC patients.

## Highlights

• The integration of bioinformatic approaches offers new insights into macrophages in hepatocellular carcinoma (HCC).
• A novel scoring system for a gene signature associated with macrophages, the Macrosig score, was constructed for HCC.
• The gene signature of macrophages could aid in the efficacy prediction of immunotherapy and molecular therapeutic regimens.

## 1 Introduction

Hepatocellular carcinoma (HCC) is the most common primary malignancy of the liver and the fourth leading cause of cancer-related mortality worldwide [1]. Over the past two decades, various studies have examined the clinical management of HCC, and novel systemic treatment options have emerged. In particular, the PD-L1 inhibitor atezolizumab plus the antiangiogenic agent bevacizumab as well as the CTLA-4 inhibitor tremelimumab-actl plus the PD-L1 inhibitor durvalumab have been approved as preferred first-line systemic therapy regimens for HCC, indicating that there is increasing support for the use of immune checkpoint inhibitor (ICI)-related regimens [[2], [3], [4]]. However, despite the considerable advances that have occurred in recent years, several questions remain unanswered: although monotherapy with PD-1- or PD-L1-related agents is generally well tolerated, the risk of immune-related adverse events is increased with combination regimens. Furthermore, the number of HCC patients who have minimal or no clinical benefit from such agents is still large. The greatest focus has been the expression of immune checkpoints on tumour cells, and although positive expression of such checkpoints can be used to identify populations who are likely to benefit clinically, testing alone is not sufficient for HCC patient selection [3]. Therefore, reliable predictors need to be identified to optimize patient benefit, minimize the risk of toxicity and guide combination approaches in patients with HCC.

As a relatively immunotherapy-resistant malignancy characterized by high cellular heterogeneity, HCC tumours are composed of a complex tumour microenvironment (TME) consisting of cellular (tumour-infiltrating immune cells and stromal cells), chemical (cytokines and chemokines), and extracellular matrix components [[5], [6], [7]]. The TME is thought to play a crucial role in HCC, and the multifaceted interactions between tumour cells and surrounding immune components in the TME can affect tumour progression, inhibit immune surveillance, and induce immune escape and tolerance by inducing the secretion of various molecules [8]. In particular, substantial clinical and experimental evidence indicates that there is a substantial level of macrophage infiltration in the TME in HCC tumours. Moreover, as fundamental immune cells in innate and adaptive immunity, macrophages play dual roles in liver cancer, and upon interacting with TME components, they are easily driven towards immunosuppressive tumour-associated macrophages (TAMs), which are strongly associated with patient survival and correlate with drug resistance [9]. In mouse models of cancer, macrophages promote cancer initiation and progression by stimulating angiogenesis, increasing the migration, invasion and intravasation of tumour cells and suppressing antitumour immunity [10]. Other studies have also suggested that macrophages are involved in tumour antigen recognition disorders, the recruitment and function of immunosuppressive cells, the secretion of immunosuppressive cytokines, immune checkpoint crosstalk, and the formation of immune privileged sites and can directly suppress T-cell responses via PD-L1 in HCC [11,12]. Notably, although macrophages express canonical markers, studies have indicated considerable transcriptomic diversity between macrophage populations even within a tissue. Given the dominant roles of macrophages in immunity, a deeper understanding of the immune cell infiltration characteristics mediated by macrophages will improve tumour immunotherapy.

Furthermore, advances in next-generation sequencing have led to an in-depth understanding of the genomics and epigenomics of HCC. Likewise, recent advances in single-cell RNA sequencing (scRNA-seq) have overcome some of the technical hurdles in the investigation of cellular heterogeneity among complex tissues such as carcinoma tissues, contributing to the identification of different subclasses of immune environments that influence tumour initiation and response and therapy. In addition, the availability of genomic datasets from public databases has made it much easier to study numerous transcriptomic and genomic profiles, providing a means to comprehensively analyze the interactions between macrophages and immune regulatory factors to predict immunotherapy response and guide treatment [13,14].

Herein, we exploit the unique multidimensional capacity of scRNA-seq to explore the landscape of immune cell infiltration in human HCCs, comprehensively delineate the multifaceted nature of macrophages, and evaluate the association between macrophage subclusters and infiltrating immune cells in the TME by integrating TCGA-liver hepatocellular carcinoma(LIHC), GEO, HCCDB and TISCH transcriptomic and genomic datasets. In this way, we first identified 13 macrophage marker genes (MMGs), based on which three distinct MMG-associated clusters were classified. Moreover, we constructed the Macrosig scoring system, which can be used as a tool to improve HCC therapy, to predict patient prognosis and response to immunotherapy and targeted drugs.

## 2 Materials and methods

### 2.1 Raw dataset acquisition

A total of five scRNA-seq datasets were acquired from TISCH (http://tisch.comp-genomics.org/) in this study. The bulk RNA-seq profile of the LIHC cohort was used as a training cohort for model development. Publicly available gene expression data and complete clinical annotations from patients with LIHC were retrospectively obtained from the HCCDB (http://lifeome.net/database/hccdb/home.html), and TCGA-LIHC data were obtained from the UCSC-Xena website (https://xenabrowser.net/datapages/). Patients without overall survival (OS) information, samples derived from borderline tumours, benign tissues or normal liver tissues and GEO samples from TCGA were excluded from this work, and a total of 574 samples with 17661 genes were included in the subsequent analyses. Furthermore, two large cohorts from the GEO database were selected as external validation cohorts. The GSE176307 dataset, which included the detailed clinical information and expression matrix of 103 patients with metastatic urothelial cancer (mUC) receiving immune checkpoint blockade (ICB) agents, was also obtained from the GEO database for further analysis [15]. In addition, data from the phase 3 JAVELIN Renal 101 trial (NCT02684006) included the complete clinical and expression profiles of advanced renal cell carcinoma (aRCC) patients receiving avelumab (anti-PD-L1) [16] and were acquired via R software.

Simple nucleotide variation (SNV) data from TCGA were also downloaded for tumour mutation burden (TMB) calculation. All RNA-seq data were converted to transcripts per million (TPM) format and further log2 transformed. The Limma and SVA algorithms were applied for raw data batch normalization [17,18].

### 2.2 Bulk RNA-seq and scRNA-seq data processing and analysis

According to the standardized pipeline, the 10X scRNA-seq data were processed through R software (version 4.2.2) via the “Seurat” package [19]. Quality control (QC) was performed on the raw matrix to obtain a high-quality scRNA-seq expression matrix for further analysis by filtering out low-quality cells according to the following criteria: 1) genes that were expressed in at least five single cells and cells that expressed more than 300 genes were selected to create a Seurat object; 2) only cells that expressed more than 300 genes and fewer than 7500 genes were included; 3) the percentage of mitochondria of each cell was calculated, and cells with more than 4 % mitochondrial genes were regarded as low-quality cells and were excluded from downstream analysis; and 4) cells with a percentage of ribosomal genes greater than 3 % and percentage of haemoglobin less than 0.1 % were included. After filtering, 22,143 genes and 59,509 cells were included. In addition, the “harmony” method in the “ScaleData” function was used to normalize the scRNA-seq data. Subsequently, the “RunPCA” function in the “Seurat” package was utilized for principal component analysis (PCA). Afterwards, uniform manifold approximation and projection (UMAP) was used for dimensionality reduction and cluster identification. Furthermore, the “FindAllMarkers” function in the “Seurat” package was used for cell cluster identification, with the parameter “resolution” set to 0.2. Then, the significant differentially expressed genes (DEGs) of each cluster were identified by calculating the log2 [fold change (FC)] value and the adjusted p value. DEGs with a |log_2_FC|≥1 and adjusted p value < 0.05 were considered MMGs of each cluster. Finally, the “SingleR” package was used for automatic cluster annotation to identify the cell types based on information from the Human Primary Cell Atlas [20].

### 2.3 Identification of MMG-associated clusters and estimation of immune cell infiltration

Unsupervised clustering was performed using the “ConsensusClusterPlus” package in R to cluster the MMGs [21] with 1000 repetitions to ensure the stability of classification. Three MMG-associated clusters were identified. The Estimation of Stromal and Immune Cells in Malignant Tumours using Expression Data (ESTIMATE) algorithm, which can be used to estimate tumour cellularity as well as tumour purity according to unique transcriptional profile characteristics, was used to calculate the ImmuneScore, StromalScore and ESTIMATE score and predict the levels of infiltrating immune cells and tumour purity [22]; furthermore, the correlations of these parameters with specific cell types were analysed by correlation analysis, with p values < 0.05 indicating statistical significance. Moreover, the Wilcox test was performed to investigate the associations between MMG subclusters and common HCC clinicopathological characteristics, including age, sex, clinical stage, T stage and survival outcome, in the TCGA cohort.

### 2.4 GSVA and functional enrichment analysis

To elucidate the functional differences in the three MMG-associated clusters obtained from the former analysis, we performed hallmark of cancer [23], Kyoto Encyclopedia of Genes and Genomes (KEGG), and Reactome enrichment analyses via data from MSigDB (https://www.gsea-msigdb.org/gsea/msigdb/) by using the “clusterProfiler” R package to analyze biological functions and comprehensively scored each gene set by GSVA to evaluate differences in biological functions between different samples at the gene and pathway levels [24,25]. We set adjusted p values < 0.05 and |logFC|>1 as the cut-off criteria, and 761 DEGs were identified. A heatmap was used to illustrate differences in pathway activity between two related subclusters.

### 2.5 Analyses of the hub genes and mutation landscapes of the MMG clusters

To clarify the prognostic value of the identified MMG clusters in HCC, we performed univariate Cox regression analysis using the 761 DEGs, and 12 hub genes among the 761 DEGs with p < 0.000001 were identified among the three MMG clusters: G6PD, MYBL2, LPCAT1, PKIB, ATP1B3, PON1, AFM, SLC1A5, SPP1, SLC22A1, ADH1C, and SMOX. These hub genes also demonstrated promising prognostic power in the Cox regression analysis. Gene mutation data for HCC patients were downloaded from the TCGA, and the TMB was calculated using the “maftools” package. We further comprehensively analysed the 12 hub genes using GSCA (http://bioinfo.life.hust.edu.cn/GSCA/#/), including analysing somatic mutation and copy number variation (CNV) profiles downloaded from the Genomic Data Commons. Somatic mutation data calculated by the R package “maftools” were sorted in mutation annotation format (MAF) format.

### 2.6 Construction of a novel Macrosig scoring system

To quantify the MMG-related profiles of individual tumours, a scoring system termed the Macrosig scoring system was developed. PCA algorithm was employed to establish the Macrosig scoring system based on the expression of the above 12 MMG-associated prognostic hub genes, and the principal components PC1 and PC2 were identified, where PC1 represents the largest proportion of the variance in the initial expression lineage to be decomposed, followed by PC2. And the sum of PC1 and PC2 served as the final Macrosig score according to the formula:

Macrosig score = ∑(PC1 + PC2)

Patients were stratified into low- and high-Macrosig Score groups at the optimal cutoff by the R package “survminer”. The correlations between the Macrosig Score and clinicopathologic variables were then examined. Decision curves were used to analyze the net benefits. A nomogram and calibration curves were employed to construct a combined prognostic diagnostic model.

### 2.7 Validation of immunotherapy response of Macrosig scoring system

To explore the correlation between the Macrosig score and immunotherapy response, a boxplot was generated to display differences in the expression of the four immune checkpoint molecules between the two Macrosig score groups. Then, We further downloaded two independent validation cohorts: GSE176307 (Metastatic Urothelial Cancer, n = 103) and NCT02684006 (advanced renal cell carcinoma, n = 886),of which all individuals received immunotherapy with transcriptomic data, complete clinical information and treatment response to evaluate the predictability and utility of the Macrosig score.

In order to validate the prognostic model, the Macrosig score for every patient in validation set was also calculated in accordance with the same formula as above mentioned, the validation samples were divided into low- and high-score groups according to the optimal cutoff value (the optimal cutoff value of the validation set was different from the threshold from the training set), and each of which was subjected to immune infiltration analysis. We confirmed the prognostic efficacy of the Macrosig score using the AUC computed by the “survivalROC” program, and Kaplan‒Meier survival curves were used to visualize the survival difference between the groups with high and low-Macrosig scores using the “survminer” package in R software, and responses between two score groups were then evaluated. Visualization of the results was performed using the “glmnet” and “pROC” packages.

### 2.8 Targeted drug response prediction of Macrosig scoring system

Next, to identify differences in the effects of targeted drugs between patients in the two groups, GSCALite was used to analyze the correlation between Macrosig score and chemotherapeutic sensitivity of distinct drugs, the half maximal inhibitory concentration (IC50) values of specific chemotherapeutic drugs commonly used for HCC treatment were estimated in the basis of ridge regression by using the package “pRRophetic” based on data from Genomics of Drug Sensitivity in Cancer (GDSC, https://www.cancerrxgene.org/).

### 2.9 Statistical analysis

All the statistical and bioinformatics analyses, data visualization and plotting were conducted using R software version 4.1.1 for Windows 64.0(http://www.R-project.org) in this study. The nonparametric Wilcoxon *t*-test was used to investigate differences in continuous categorical variables between the two groups. To compare three or more groups, Kruskal- Wallis (KW), one-way ANOVA, and Welch one-way ANOVA were applied. Comparisons of proportions were performed with the chi-square test or Fisher's exact test. Cox regression analysis was used to identify prognostic genes. Pearson's correlation coefficients was used to assess correlations between two continuous variables. Survival analysis was performed by the Kaplan‒Meier method. Permutation test was used to examine the correlation between the Macrosig score and mutant frequency. Independent prognostic analysis was conducted by univariate and multivariate Cox proportional hazard regression, estimating hazard ratio (HR) and 95 % confidence interval (CI) simultaneously.Unless otherwise noted, p < 0.05 was set as the significance threshold(*p < 00.05,**p < 00.01, ***p < 00.001, ***p < 00.0001).The Benjamini–Hochberg method was used to adjust the p value for multiple tests using the R function “p.adjust”. Data in this study are all presented as the mean ± SEM from three independent experiments unless otherwise noted.

## 3 Results

### 3.1 Study design and data processing

The workflow of the study was displayed in Fig. 1. Various packages of R software were employed for analyses. First, we obtained HCC-related scRNA-seq datasets from the TISCH database and performed cell clustering and cell type annotation by using the Seurat, Harmony, ScaleData, RunUMAP, FindAllMarkers, SingleR R packages and the CellTypist Python package. Second, we downloaded HALLMARK(v7.5.1) from Msigdb, and we utilized GSVA R package to analysis the enrichment scores of different single cell data. Second, we obtained bulk RNA-seq profiles related to HCC from the UCSC-Xena and HCCDB database and merged them using the FactoMineR and factoextra R package,and obtaind 13 macrophage marker genes (MMGs) by Cox univariate analysis. Subsequently, Unsupervised clustering was performed using the “ConsensusClusterPlus” package in R to cluster the MMGs into 3 macrophage-related clusters, The ssGSEA function of GSVA R-package was used to evaluate immune infiltration. Third, we identified 12 hub genes based on differentially expressed genes of 3 clusters by univariate regression analysis. To quantify the MMG-related profiles of individual tumours, a scoring system termed the Macrosig scoring system was developed by using PCA algorithm. Last, we performed prognostic analysis using survival R packages and pRRophetic package for external datasets validation.

**Fig. 1:**
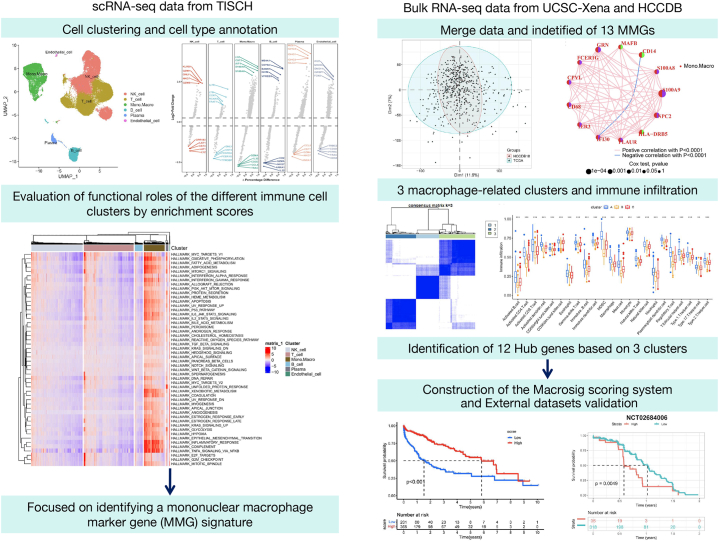
Analysis flow chart.

### 3.2 The landscape of immune cell infiltration in HCC as assessed by scRNA-seq analysis

Based on scRNA-seq data from GSE140228 [26], we obtained gene expression profiles of 59509 high-quality cells from sixteen patients who were pathologically diagnosed with liver cancer, including thirteen males and three females, and these cells were identified for subsequent analysis after strict quality control. Fourteen cell clusters were identified by dimensionality reduction analysis with UMAP. Subsequently, the cell identity of each cluster was annotated using a reference dataset from the Human Primary Cell Atlas, and six cell types were identified: natural killer (NK) cells, T cells, mononuclear macrophages, B cells, plasma cells and endothelial cells (Fig. 2A). Next, the proportions and relationships of different cell types in each cluster were identified, as displayed in Fig. 2B; T cells, NK cells and mononuclear macrophages were the three most predominant cell types in HCC. The FindAllMarkers function was used to detect DEGs in the different cell types. The top 5 genes with high or low expression ranked according to the log_2_FC value for each of the six types of cells are shown in Fig. 2C, and all the differentially expressed marker genes are listed in Table S1.

**Fig. 2:**
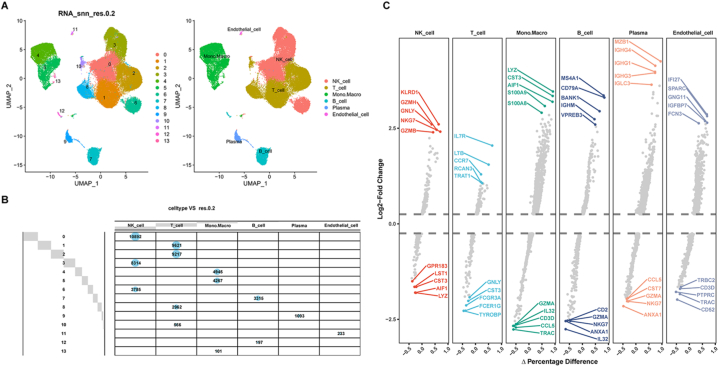
scRNA-seq analysis to identify different immune cell populations in HCC. **a** UMAP analysis was adopted to identify 14 different clusters and 6 cell types from the collected samples. **b** Proportions and relationships of different cell types in each cluster. **c** Volcano plot showing the top 5 marker genes with high or low expression in each cell cluster.

We further conducted GSVA using the hallmark gene sets as reference gene sets derived from MSigDB to determine the enrichment scores of the different immune cell clusters to provide insights into the different biological functions of the groups. Excitingly, we found that mononuclear macrophages showed enrichment of the following hallmarks: inflammatory response, complement, TNF-α signalling via NF-κB, oxidative phosphorylation, adipogenesis and mTORC1 signalling; thus, these cells had a promising antitumour immunophenotype (Fig. 3). Therefore, in this study, we focused on identifying a mononuclear macrophage marker gene (MMG) signature and further investigated the relationships between different immune subtypes and molecular subtypes.

**Fig. 3:**
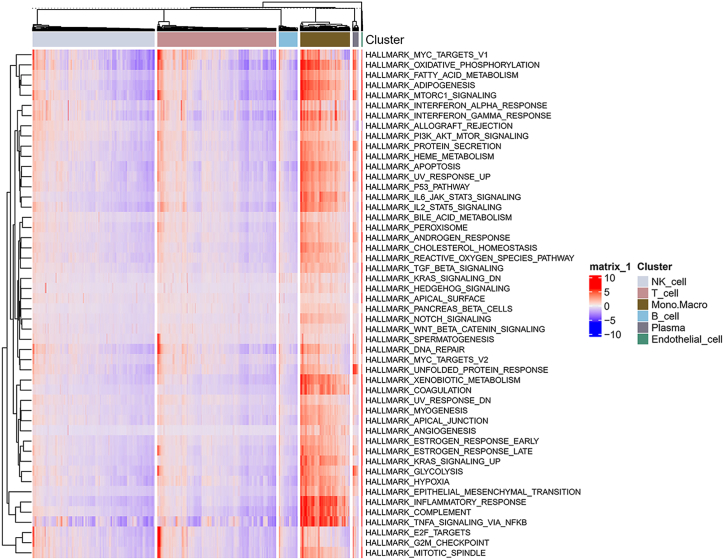
Heatmap of the results of GSVA to explore the functional roles of different immune cell clusters. The hallmark gene set (v7.5.1) was downloaded from MSigDB, and the R GSVA package was used to calculate the enrichment scores of different immune cell clusters based on single-cell data.

### 3.3 Identification of MMG expression profiles in HCC

To unravel the underlying biological characteristics of macrophage immunophenotypes, we identified a total of 17661 genes from 574 samples from the TCGA expression profile dataset, which included 371 tumour samples, and from the HCCDB18 dataset, which included 203 tumour samples. The limma and SVA algorithms were applied to the data for batch correction, and two datasets were separated in the PCA plots because of heterogeneity, which was eliminated after normalization (Fig. 4A). Next, we selected the top 50 MMGs with high expression in macrophages identified by the abovementioned scRNA-seq analysis, among which 48 genes were expressed in the combined data. Thirty-two of the 48 MMGs showed a significant effect on patient survival outcomes in the combined dataset (Fig. S1). Ultimately, we obtained 13 MMGs in HCC according to the cut-off criteria of |logFC|>1 and adjusted p value < 0.05 in the univariate Cox regression analysis (Table S2): S100A9, CD14, GRN, NPC2, CD68, IER3, MAFB, HLA-DRB5, CPVL, IFI30, PLAUR, S100A8, and FCER1G; furthermore, the comprehensive landscape of MMG interactions, regulatory connections, and prognostic value in patients with HCC is shown in Fig. 4B. We next analysed the expression distribution of these 13 MMGs, and as expected, they were mainly expressed in the macrophage cluster (Fig. 4C). In addition, we combined multiple single-cell liver cancer datasets from TISCH to verify the above conclusion and confirmed that the 13 identified MMGs were generally highly expressed in macrophages (Fig. S2).

**Fig. 4:**
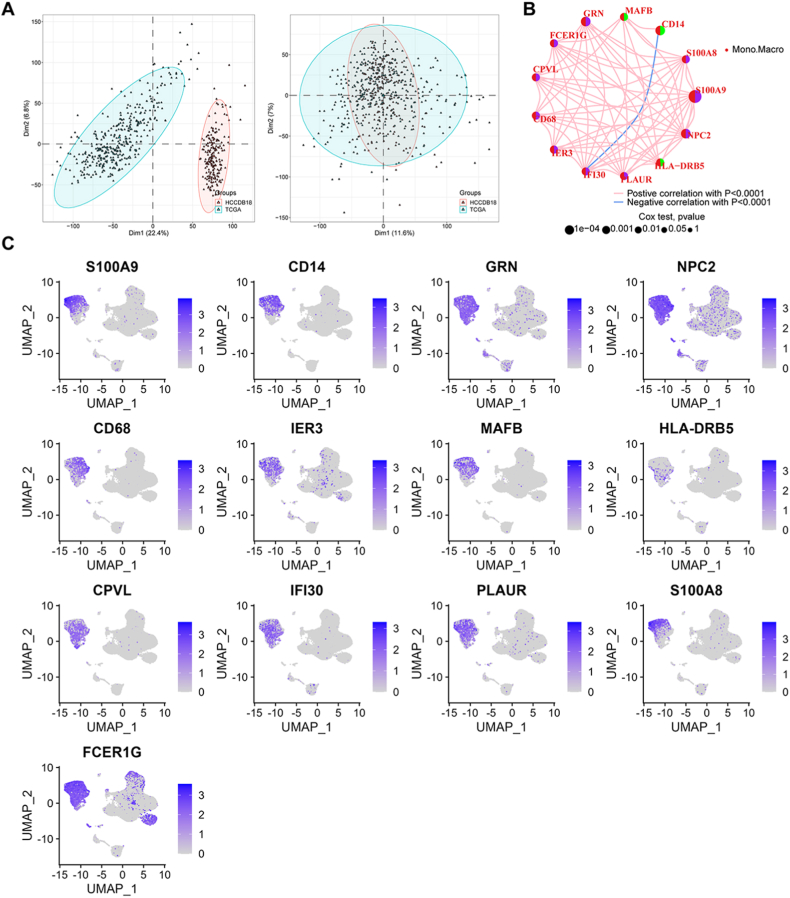
Identification of MMG expression profiles in HCC. **a** TCGA and HCCDB18 HCC data were merged, and the R packages “limma” and “sva” were used to remove batch effects; the samples were pooled before (left) and after (right) batch effect removal. **b** Thirteen macrophage marker genes of HCC and the gene–gene communication network of the marker genes. Purple indicates a prognostic risk factor, and green indicates a protective factor. **c** Thirteen macrophage marker genes were mainly specifically expressed in the macrophage cluster, as expected. (For interpretation of the references to colour in this figure legend, the reader is referred to the Web version of this article.)

### 3.4 Three MMG-associated clusters and their functional differences

To further explore the expression profiles of tumour-infiltrating MMGs in HCC, we applied an unsupervised consensus clustering algorithm to categorize patients with HCC based on the expression of the 13 MMGs. Our results showed that k = 3 was the optimal selection value for sorting the entire cohort into 3 clusters: A (n = 145), B (n = 194) and C (n = 227) (Fig. 5A). Furthermore, we explored the prognostic implications of macrophage gene clusters by integrating them with survival information. K‒M plotter analysis revealed that patients in gene cluster B had a better prognosis, whereas patients in gene clusters A and C had unfavourable outcomes (log rank test, p = 0.028; Fig. 5B). In addition, we found that the expression levels of MMGs were mostly lower in cluster B than in clusters A and C, except for that of CD14 (Fig. 5C). Considering the role of macrophage subtypes in HCC, we also compared the clinical features of the 3 clusters of macrophages. The relationships between HCC patient clinical factors and macrophage subtypes were delineated in a heatmap (Fig. 5D).

**Fig. 5:**
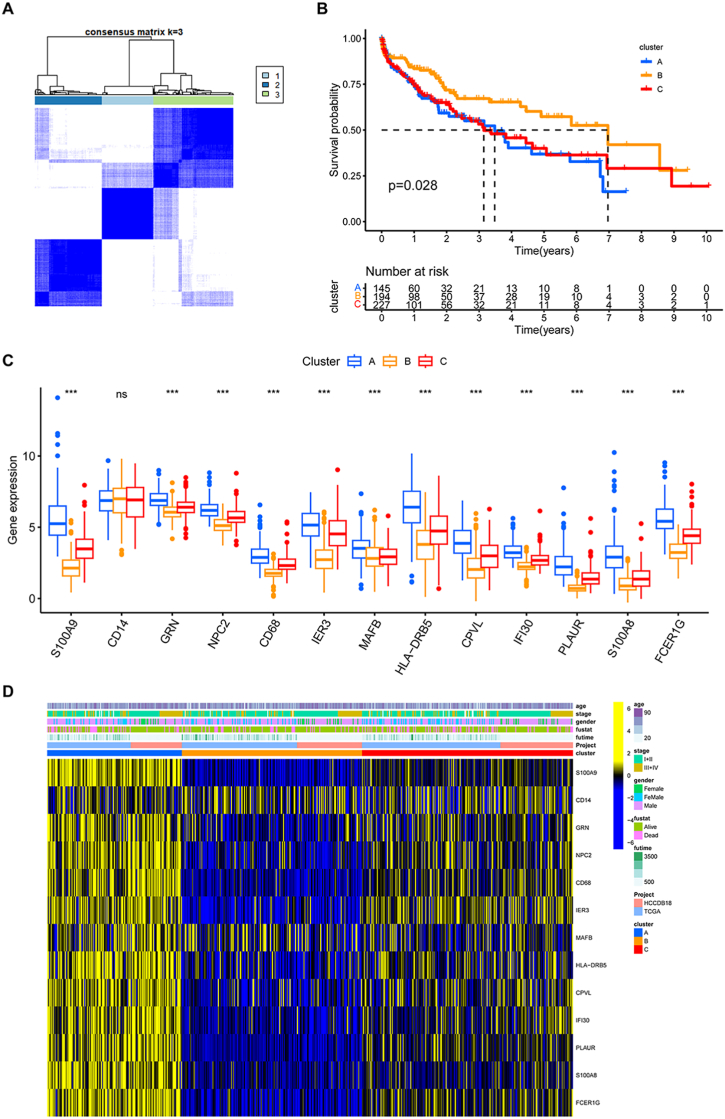
Identification of three MMG-associated subclusters. **a**n Unsupervised clustering analysis based on the 13 MMGs. **b** KM survival curve for each MMG-associated subcluster. **c** Expression of the MMGs in different subclusters. **d** Heatmap showing the differences in clinicopathologic features and the expression levels of MMGs among the 3 subclusters. (* represents p < 0.05, ** represents p < 0.01, *** represents p < 0.001, ns represents p > 0.05).

To identify differentially enriched pathways among the 3 macrophage clusters in the HCC cohort, we downloaded the hallmark, KEGG and Reactome pathway sets from MSigDB and carried out GSVA to identify the enriched pathways of these 3 clusters. The enrichment heatmap illustrated that cluster B had significant enrichment of inflammatory and antitumour hallmarks (including PI3K-AKT-mTOR signalling, IFN-γ response, IFN-α response, IL-6-JAK-STAT3 signalling, IL-2-STAT5 signalling, Kras signalling, TNF-α signalling via NF-κB and inflammatory response). Cluster B had significant enrichment of KEGG pathways related to immune infiltration (including the NOD-like receptor signalling pathway, Toll-like receptor signalling pathway, T-cell receptor signalling pathway, B-cell receptor signalling pathway, cytokine‒cytokine receptor interaction pathway and chemokine signalling pathway). For the Reactome pathway, cluster B had significant enrichment of inflammation and immune activity (including PD-1 signalling, DAP12 interactions, IL-10 signalling, and signalling by interleukins and inflammasomes) (Fig. S3).

### 3.5 Estimation of TME infiltration and functional differences in distinct macrophage clusters

**Fig. 6:**
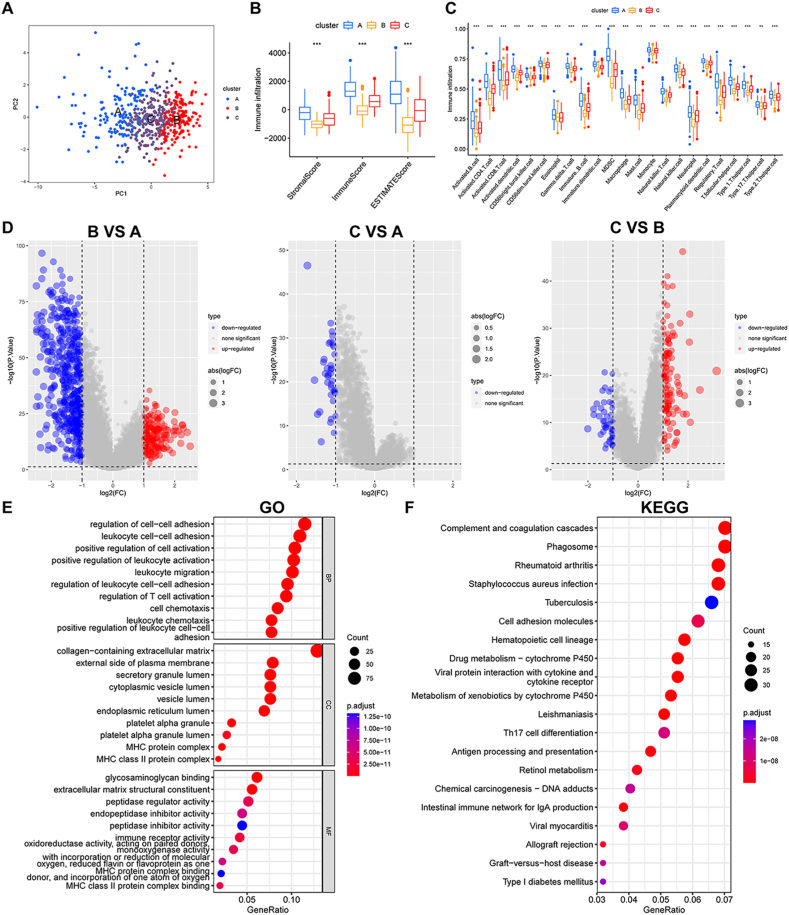
Estimation of TME infiltration and functional differences among the three macrophage clusters. **a** PCA diagram showing the distribution of different subclusters. **b** Differences in the stromal score, immune score and ESTIMATE score among the three different clusters. **c** Comparisons of immune cell infiltration among different clusters. **d** Differentially expressed genes (DEGs) among the three clusters are shown in volcano plots. **e, f** GO and KEGG analyses were conducted, and the results were visualized. (* represents p < 0.05, ** represents p < 0.01, *** represents p < 0.001, ns represents p > 0.05).

Macrophages play a vital role in antitumour immunity. Although a close relationship between macrophage subclusters and immune activity was identified, macrophages and the immune response are processes involving multiple steps and molecules that act in a highly united and cooperative manner. We further explored the relationships between macrophage subclusters and immune cell infiltration in HCC patients. PCA was used for dimensionality reduction (Fig. 6A), and by using the ESTIMATE algorithm, we found that cluster B had lower stromal scores, immune scores and ESTIMATE scores than clusters A and C (p < 0.001) (Fig. 6B). To investigate the role of MMGs in the TME of HCC, we assessed the correlations between the 3 macrophage clusters and 23 human immune cell subsets using single-sample gene set enrichment analysis (ssGSEA). Notably, we observed significant differences in the infiltration of most immune cells among the three clusters; for example, cluster A was found to have greater immune cell infiltration, but cluster B had less immune infiltration (Fig. 6C). Next, we performed differential gene analysis for the three clusters, and DEGs among the three clusters were visualized in volcano plots. As shown in Fig. 6D, the number of DEGs between cluster B and cluster A was the greatest, which was consistent with the above phenotypic results. On this basis, the gene sets were filtered using the criteria |logFC|> 1 and p < 0.05, and 761 DEGs were acquired. The clusterProfiler R package was used for GO analysis. The 761 DEGs were mainly enriched in the biological process (BP) terms cell‒cell adhesion, leukocyte activation, and cell chemotaxis; the cellular component (CC) terms collagen-containing extracellular matrix, external side of plasma membrane and vesicle lumen; and the molecular function (MF) terms glycosaminoglycan binding, extracellular matrix structural constituent and immune receptor activity (Fig. 6E). Further KEGG pathway analysis revealed that the DEGs were mainly enriched in complement and coagulation cascades, phagosomes, drug metabolism (cytochrome P450), and metabolism of xenobiotics by cytochrome p450, which may imply that the macrophage subclusters have effects on antitumour drug metabolism (Fig. 6F).

### 3.6 Hub gene subtypes based on macrophage clusters and construction of the Macrosig scoring system

**Fig. 7:**
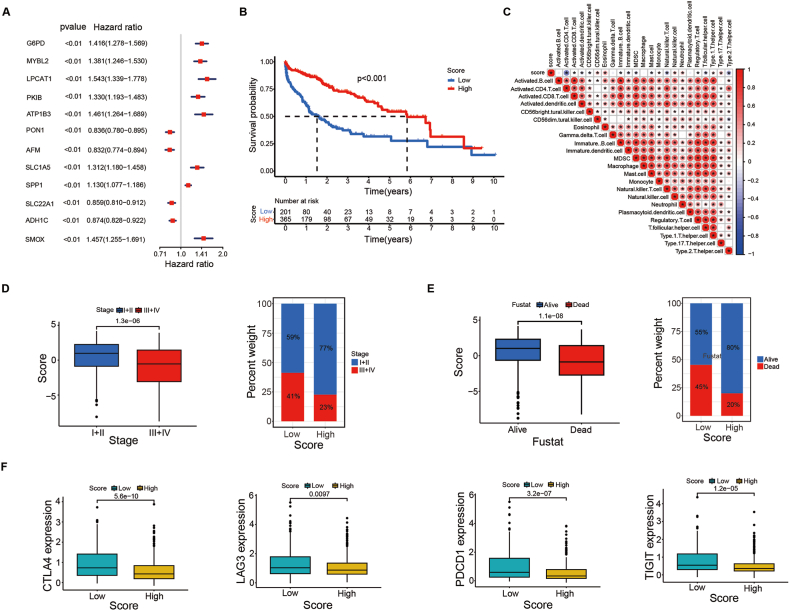
Macrophage cluster hub genes and construction of the Macrosig scoring system. **a** 12 of 761 hub DEGs among the three macrophage clusters demonstrated notable prognostic power in the Cox regression analysis. **b** Using PCA, the Macrosig scoring system was developed based on 12 genes, and survival analysis was performed between groups with high and low scores. **c** Correlation with immune cell infiltration. The size and colour of the circles represent the Pearson correlation coefficients. A red circle indicates a positive correlation, and a blue circle indicates a negative correlation. One asterisk (*) indicates a p value less than 0.05 (p < 0.05). **d** Correlation between disease stage and the Macrosig score. **e** Correlation between survival outcome and the Macrosig score. **f** Expression of immune checkpoint genes (including CTLA-4, LAG3, PDCD1 and TIGIT) in the high- and low-score groups. (For interpretation of the references to colour in this figure legend, the reader is referred to the Web version of this article.)

Multiple studies have shown that the immune system is related to therapeutic outcomes, and protumour or antitumour activity can exist [27,28]. To clarify the prognostic value of the identified macrophage clusters in HCC, we performed a univariate Cox regression analysis using the 761 DEGs, and 12 hub genes of the 761 DEGs with p < 0.000001 were identified among the three macrophage clusters (G6PD, MYBL2, LPCAT1, PKIB, ATP1B3, PON1, AFM, SLC1A5, SPP1, SLC22A1, ADH1C, and SMOX), which also demonstrated noticeable prognostic power in the Cox regression analysis (Fig. 7A, Table S3). In addition, we also conducted a comprehensive analysis of 12 genes using the GSCA datasets, and they were all differentially expressed in the HCC TCGA dataset; we also analysed deleterious mutations, mutation distribution, CNV percentage, and methylation differences (Fig. S4).

Given the crucial regulatory function of macrophages in the evaluation of prognosis and the TME immune landscape, we further constructed a scoring system with PCA (PC1+PC2) based on the 12 hub DEGs. We termed this the Macrosig scoring system. We divided patients into low-score and high-score groups to quantify macrophage regulation and the immune microenvironment in individual HCC samples. A higher Macrosig score indicated a longer survival time (p < 0.001) (Fig. 7B). Moreover, according to the tumour-infiltrating immunocyte analysis, we determined the levels of 23 types of infiltrating immune cells in patients with different Macrosig scores and found that the Macrosig score was negatively correlated with the majority of infiltrating immune cells (Fig. 7C). These results preliminarily suggest that the greater the Macrosig score is, the less immune cell infiltration there is and the better the prognosis of HCC patients.

### 3.7 Correlations between the Macrosig score, immunological characteristics and clinical features

In terms of clinicopathological features, the Macrosig score was also correlated with patient stage and survival status. As shown in Fig. 7D, significant differences in the Macrosig score were observed among different HCC stages; patients with a low-Macrosig score (59 % stage I + II and 41 % stage III + IV) presented more severe stages of disease than those with a high Macrosig score (77 % stage I + II and 23 % stage III + IV) (p < 0.001). More importantly, we also observed that the high-Macrosig score group (80 % alive and 20 % dead) presented a prominent survival advantage over the low-Macrosig score group (55 % alive and 45 % dead) (p < 0.001, Fig. 7E), indicating that the Macrosig score has potential prognostic value for HCC patients. Furthermore, we investigated the correlation between the Macrosig score and immune checkpoints. In line with the above negative correlation of the Macrosig score with the levels of most infiltrating immune cells, the results showed that patients with a low-Macrosig score had significantly greater expression of most immune checkpoint genes, including CTLA-4, LAG3, PDCD1 and TIGIT (Fig. 7F), than patients with a high score, which suggested that HCC patients in the low-Macrosig score group were more likely to respond to immunotherapy.

**Fig. 8:**
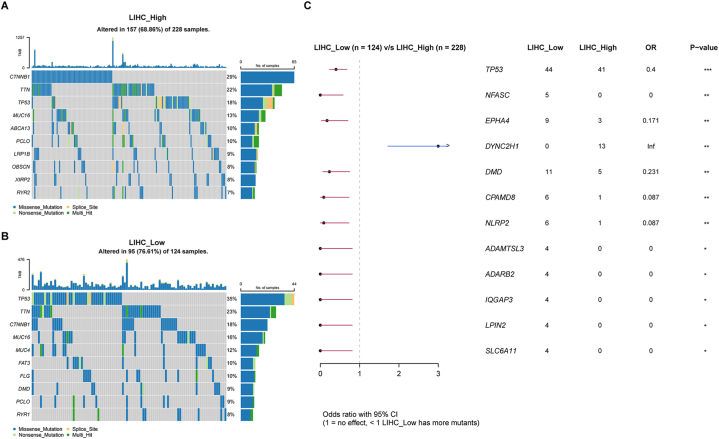
Somatic variants in HCC driver genes in the low- and high Macrosig score groups. **a, b** Somatic mutation features in the low- and high Macrosig score groups are displayed in a waterfall plot. TMB: tumour mutation burden. **c** Differences in somatic mutation frequency were assessed by maftools.

Increasing evidence has demonstrated that somatic mutation patterns are associated with responsiveness to immunotherapy. We thus assessed the distribution of somatic variants in HCC driver genes between the low- and high Macrosig score subgroups. The HCC driver genes were identified by using the maftools R package [29], and the top 10 driver genes with the highest alteration frequency in the high (altered in 157 of 228 samples) and low (altered in 95 of 124 samples) Macrosig score groups were further analysed. The mutation annotation files revealed that the alteration frequencies of TP53, NFASC, EPHA4, DMD, CPAMD8, NLRP2, ADAMTSL3, ADARB2, IQGAP3, LPIN2 and SLC6A11 were significantly greater in the low-score group than in the high-score group, but this pattern did not hold for DYNC2H1 (Fig. 8A–C). These results might provide novel ideas for investigating the mechanism of gene mutation in immune checkpoint blockade therapy.

### 3.8 The relationship between the Macrosig score and cytokine levels

**Fig. 9:**
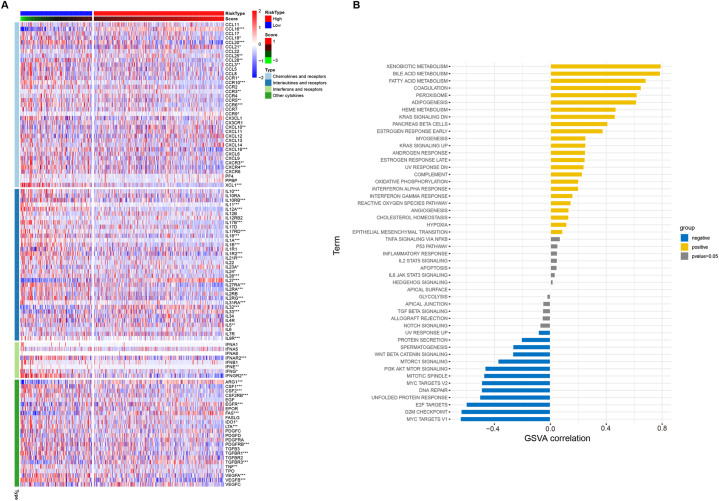
Relationships of the Macrosig score with cytokine expression and hallmark pathways. **an** Immune activity-related cytokine expression differed significantly between the high- and low-Macrosig score groups. **b** The correlations between the Macrosig score and 50 hallmark terms were calculated, and ggplot2 was used to plot the results.

Chemokines, interleukins and interferons are all cytokines; they play important roles in the composition of the TME, coordinate immune cell trafficking, exert multifaceted roles in the tumour immune response and tumour biology, and have a strong influence on patient prognosis and response to therapy; therefore, the cytokine network has emerged as a potential immunotherapy target [[30], [31], [32]]. TAMs generally include two subtypes: M1 macrophages play an antitumour immune role, while M2 macrophages promote tumour growth, invasion and migration and inhibit tumour immunity by secreting anti-inflammatory cytokines. However, whether the Macrosig score has an effect on cytokine expression is unknown. We next examined the expression of various chemokines in the high- and low-Macrosig score groups and observed that the expression of most immune activity-related cytokines significantly differed between the groups with different Macrosig scores, with the high-score group expressing decreased levels of cytokines (Fig. 9A). In the subsequent analysis, the correlations between the Macrosig score and 50 hallmark terms were calculated, and the results were visualized via ggplot2 (Fig. 9B). The Macrosig score was positively correlated with xenobiotic metabolism, bile acid metabolism, fatty acid metabolism, and so on and negatively correlated with MYC targets V1, the G2M checkpoint, E2F targets, the unfolded protein response, the unfolded protein response, DNA repair, and so on.

### 3.9 Macrosig score-related treatment strategy for HCC

**Fig. 10:**
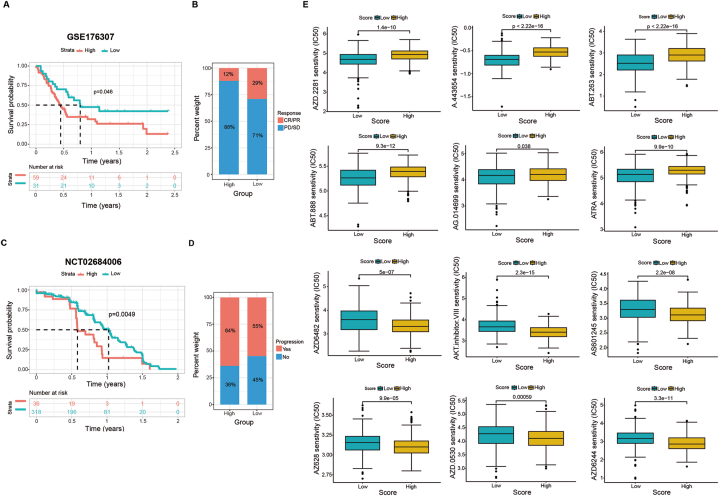
Verification of the efficacy of the Macrosig score in predicting immunotherapy response and sensitivity to targeted therapy with an expanded dataset. **a-d** Differences in antitumour immunotherapy responsiveness and survival probability between the low- and high Macrosig score groups in GSE176307 and NCT02684006. **e** We comprehensively evaluated the sensitivities to different targeted drugs in the high and low-Macrosig score groups using pRRophetic.

Immune checkpoint blockade therapy, which is used to block T-cell inhibitory molecules in cancer treatment, has shown promising outcomes and the potential to improve disease conditions in advanced cancers, but it is not effective in all patients [33,34]. The higher the Macrosig score was, the lower the immune infiltration, immune checkpoint expression, cytokine expression and frequency of gene mutations. Therefore, we hypothesized that patients in the high-score group would be insensitive to immunotherapy. To test this hypothesis, we used an immunotherapy database to verify the efficacy of the Macrosig score in predicting immunotherapy response. By employing a real-world dataset derived from patients with metastatic urothelial cancer treated with immune checkpoint inhibitors (GSE176307) [15], we discovered that the survival probability was significantly greater in the low-Macrosig score group (p = 0.046); moreover, the complete response (CR) or partial response (PR) rate was also greater than that in the high-Macrosig score group (29 % CR + PR vs. 12 % CR + PR) (Fig. 10A and B). In addition, based on data from the phase 3 JAVELIN Renal 101 trial (n = 886; NCT02684006) that aimed to evaluate the efficacy of avelumab (an anti-PDL1 antibody) [16], we further revealed that patients with low-Macrosig scores exhibited longer survival times and lower disease progression rates than those with high Macrosig scores (p = 0.0049) (Fig. 10C and D); all of these results demonstrated that the Macrosig score can play a crucial role in predicting the immune response.

Regarding targeted therapy, we comprehensively evaluated the sensitivities of targeted drugs in different Macrosig score groups using pRRophetic, with a higher IC50 indicating less treatment sensitivity. The results showed that patients in the low-score group had lower IC50 values for some targeted therapeutics, including AZD.2281 (olaparib), A.443654 (an Akt inhibitor), ABT.263 (navitoclax), ABT.888 (a PARP1 inhibitor), AG.014699 (rucaparib) and ATRA; that is, patients in the low-Macrosig score group were more likely to benefit from these drugs. The IC50 values of AZD6482 (a PI3Kβ inhibitor), AKT inhibitor VIII, AS601245 (a JNK inhibitor), AZ628 (a Raf kinase inhibitor), AZD.0530 (saracatinib), and AZD6244 (selumetinib) were significantly lower in the patients in the high-Macrosig score group than in those in the low-Macrosig score group (Fig. 10E), suggesting that patients with high Macrosig scores are more likely to be sensitive to these treatments. These results indicate that the Macrosig score can be used to predict sensitivity to targeted drugs and help optimize treatment options for HCC patients. In addition, we used the RegNetwork database to predict the upstream miRNAs and transcription factors of the above 12 genes (Fig. S5) to establish a foundation for further mechanistic studies.

## 4 Discussion

HCC is among the tumour types that are resistant to immune checkpoint inhibition, with only a minority of HCC patients benefiting. Identifying reliable predictors to optimize patient benefit, minimize the risk of toxicity and guide combination approaches for HCC patients remains a challenge. Mounting evidence has demonstrated that macrophages are highly enriched in the tumour immune microenvironment and play an indispensable role in inducing immunosuppression in liver malignancies [35,36]. Previous studies have verified that macrophages act as key orchestrators associated with cancer progression and distant metastasis and that their abundance is correlated with clinical prognosis; for example, macrophages can promote the expression of PD-L1 on HCC cells through cytokines or directly suppress T-cell responses via PD-L1, and cytokines secreted by macrophages can affect TAM-mediated phagocytosis via the STAT3 signalling pathway, resulting in changes in overall survival and recurrence-free survival [11,37,38]. However, there is still significant room for improvement in macrophage-mediated immune modulation, and comprehensive molecular analysis of macrophages in HCC is relatively lacking [39,40].

On the basis of existing research, herein, we carried out a comprehensive bioinformatic analysis of the transcriptomic landscape of human HCCs based on scRNA-seq and bulk RNA sequencing data. In the present study, we obtained 13 MMGs (S100A9, CD14, GRN, NPC2, CD68, IER3, MAFB, HLA-DRB5, CPVL, IFI30, PLAUR, S100A8, FCER1G), all of which are mainly specifically expressed in the macrophage cell cluster and correlated with the prognosis of HCC patients; this finding may provide insights into the molecular mechanisms for further clinical studies to improve antitumour therapy efficacy in HCC. Next, we identified 3 distinct MMG-associated clusters (clusters A, B and C) and evaluated the association between these subclusters and infiltrating immune cells in the TME. We observed that cluster B patients had more favourable outcomes than those in clusters A and C. As expected, cluster B was significantly characterized by enrichment of inflammatory and immune-related biological behaviours and pathway terms, including the NOD-like receptor signalling pathway, Toll-like receptor signalling pathway, T-cell receptor signalling pathway, B-cell receptor signalling pathway, cytokine‒cytokine receptor interaction and chemokine signalling pathway, PD-1 signalling, DAP12 interactions, and signalling by interleukins and inflammasomes. We also found that cluster B had a lower stromal score, immune score and ESTIMATE score, in line with a previous study showing that a high immune infiltration score was associated with a poor prognosis, confirming the reliability of our classification of immune phenotypes for the different clusters [41].

Moreover, 761 DEGs were identified among the three clusters, and functional enrichment analysis revealed that the DEGs were enriched mainly in the terms complement and coagulation cascades, phagosome, drug metabolism (cytochrome P450), and metabolism of xenobiotics by cytochrome p450, implying that the macrophage subclusters may affect antitumour drug metabolism. Twelve hub genes (G6PD, MYBL2, LPCAT1, PKIB, ATP1B3, PON1, AFM, SLC1A5, SPP1, SLC22A1, ADH1C, and SMOX) were further assessed. Given the crucial regulatory function of macrophages in prognosis and the potential effects of macrophage subclusters on antitumour drug metabolism identified in this study, a comprehensive evaluation of macrophage subtypes will enhance our understanding of TME cell infiltration. Moreover, we established a reliable and effective system termed the Macrosig scoring system to evaluate macrophages in individual HCC patients. Notably, we found this system to be an efficient prognostic tool that can guide precision immunotherapy and targeted drug therapy. Our research lays a foundation for a deeper understanding of patient antitumour immune response and provides guidance for more individualized and effective combination therapy regimens related to the Macrosig score.

Despite the promising results obtained in this study, however, there are still some inevitable limitations and shortcomings that should be addressed in future studies: Although the accuracy of transcriptome data combining scRNA-seq and bulk RNA-seq data is relatively high, the sample size of the scRNA-seq data was limited, and differences in sequencing platforms could introduce variations in sequencing errors, which can be biological, systematic or random. More advanced techniques and tools for data analysis are needed to uncover genuine biological differences between samples and develop stationary model-building methods in future studies. Moreover, analyses based on public databases may generate biases in prediction results, entailing further validation by using real-world data from HCC human tissues and cell lines, and additional better-designed prospective studies should be performed to enhance the understanding of this topic.

This study provided a novel macrophages-based prognostic model which might reflect the immune infiltration state and can be used to predict sensitivity to immunotherapy or different targeted drugs and help optimize treatment options for HCC patients. Our findings also provided genomic evidence for future research directions on macrophages related therapeutic strategies, and contribute to developing precision medicine for HCC patients. Although this research is mainly based on bioinformatics analysis, it is an innovative and pioneering study, which could help to be assistance in future clinical practice.

## 5 Conclusions

In conclusion, in this study, the immune microenvironment landscape of human hepatocellular carcinoma (HCC) and distinctive macrophage marker genes (MMGs) were comprehensively delineated with multiple single-cell and bulk RNA sequencing datasets. Additionally, a deeper analysis of macrophages based on machine learning revealed three MMG-associated clusters and 12 hub genes. Furthermore, a novel scoring system named the Macrosig score was constructed, characterized by differences in immune infiltration, mutation frequency and immune checkpoint expression, which has the potential to aid in the prediction of immunotherapy efficacy and personalization of molecular therapeutic regimens.

## Data availability statement

The scRNA-sequencing datasets generated in our study were acquired from TISCH (http://tisch.comp-genomics.org/). Publicly available gene expression data and complete clinical annotations from patients with LIHC were retrospectively obtained from the HCCDB (http://lifeome.net/database/hccdb/home.html), and TCGA-LIHC data were obtained from the UCSC-Xena website (https://xenabrowser.net/datapages/).Data and code associated in this article has been deposited into a publicly available repository (Mendeley Data, Reserved https://doi.org/10.17632/4t5mtrv49r.1)

## Ethical approval

Approval by an ethics committee was not needed for this study because these data is from public data platform.

## Consent for publication

Not applicable.

## Funding

This work was supported by 10.13039/501100001809National Natural Science Foundation of China (Grant Nos. 82102761), 10.13039/501100013105Shanghai Rising-Star Program (Grant Nos. 23YF1441200), Fudan university 10.13039/501100010108Zhongshan Hospital Youth Fund (Grant Nos. 2021ZSQN49).

## CRediT authorship contribution statement

**Tong Li:** Writing – review & editing, Writing – original draft, Project administration, Methodology, Investigation, Funding acquisition, Formal analysis, Data curation, Conceptualization. **Xin Xu:** Writing – review & editing, Visualization, Validation, Supervision, Resources, Methodology, Formal analysis, Data curation. **Mengzhou Guo:** Writing – review & editing, Validation, Supervision, Software, Resources, Methodology, Funding acquisition. **Jing Guo:** Writing – review & editing, Supervision, Project administration. **Kiyoko Nakayama:** Writing – review & editing, Resources, Investigation. **Zhenggang Ren:** Writing – review & editing, Validation, Supervision. **Lan Zhang:** Writing – review & editing, Visualization, Supervision, Project administration, Formal analysis, Conceptualization.

## Declaration of competing interest

The authors declare that they have no known competing financial interests or personal relationships that could have appeared to influence the work reported in this paper.

## Figures and Tables

**figs1:**
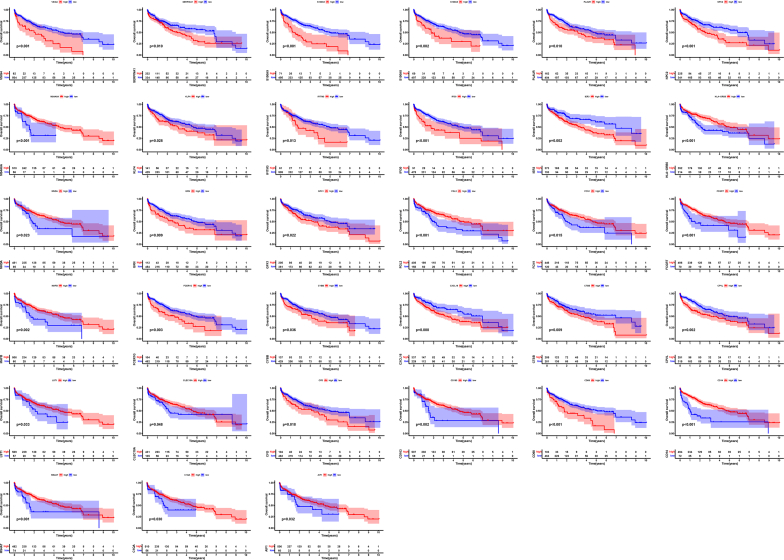


**figs2:**
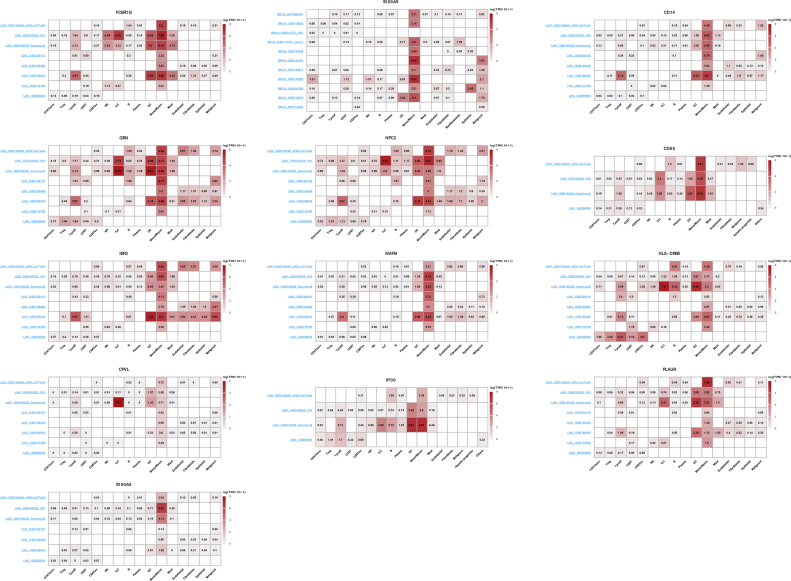


**figs3:**
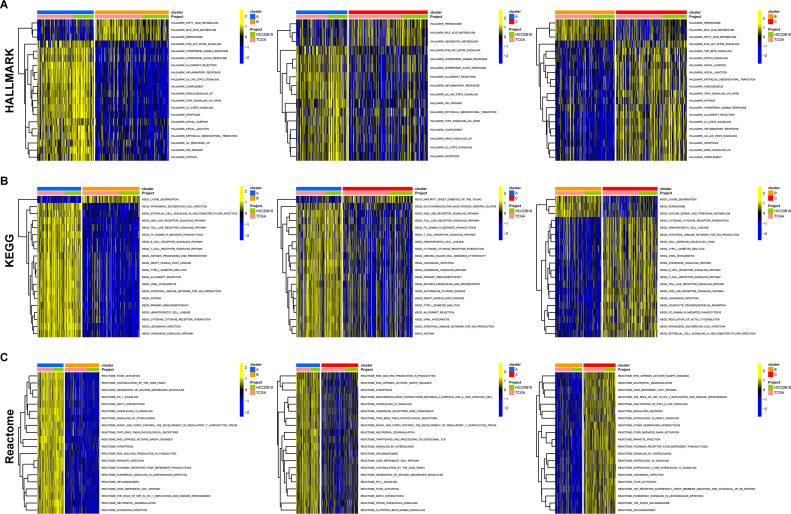


**figs4:**
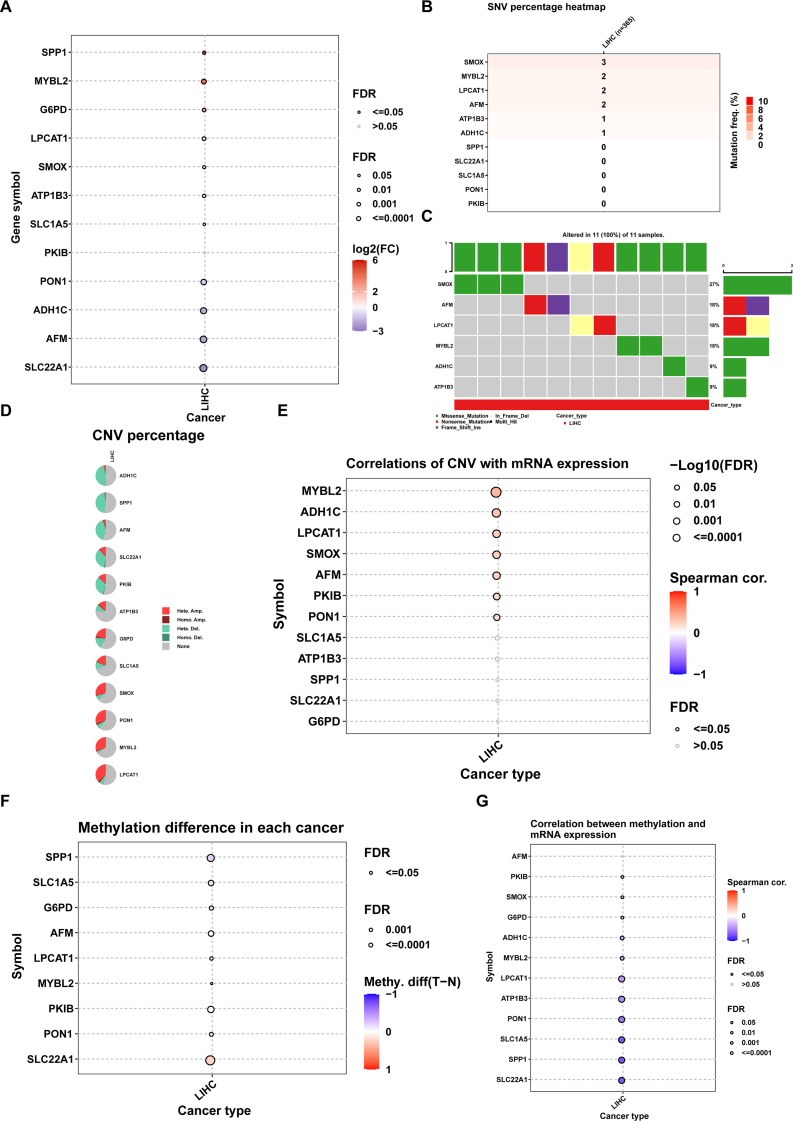


**figs5:**
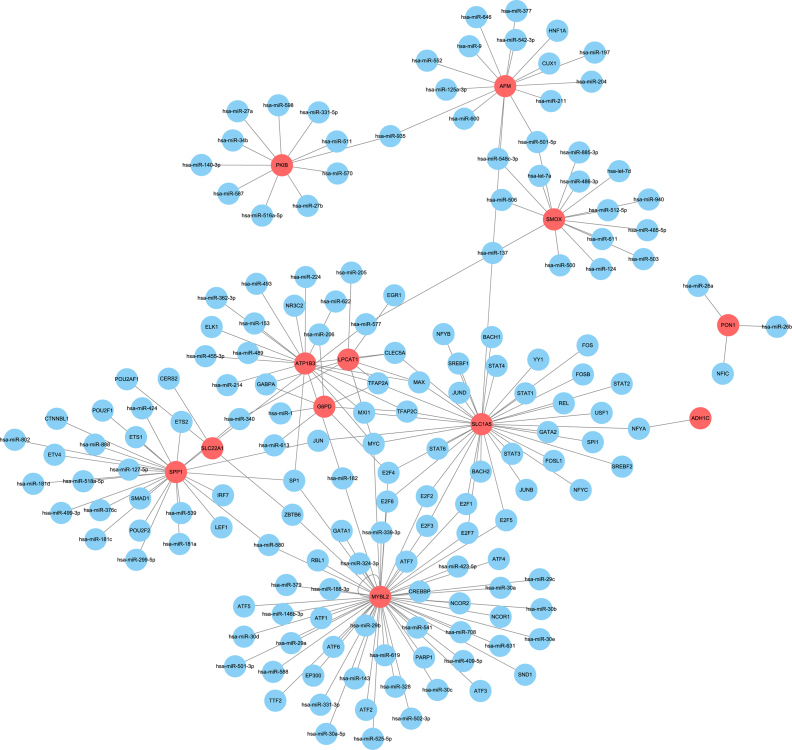


## Abbreviations

HCC: Hepatocellular carcinoma
LIHC: Liver hepatocellular carcinoma
TME: Tumour microenvironment
TAM: Tumour-associated macrophage
CNV: Copy number variation
TMB: Tumour mutation burden
TCGA: The Cancer Genome Atlas
GEO: Gene-Expression Omnibus
DEGs: differentially expressed genes
UMAP: Uniform manifold approximation and projection
PCA: Principal component analysis
GSVA: Gene set variation analysis
ssGSEA: Single-sample gene-set enrichment analysis
ICIs: immune checkpoint inhibitors
MMGs: Macrophage marker genes
